# Cerebrospinal fluid liquid biopsy for detecting somatic mosaicism in brain

**DOI:** 10.1093/braincomms/fcaa235

**Published:** 2021-01-21

**Authors:** Zimeng Ye, Zac Chatterton, Jahnvi Pflueger, John A Damiano, Lara McQuillan, A Simon Harvey, Stephen Malone, Hongdo Do, Wirginia Maixner, Amy Schneider, Bernadette Nolan, Martin Wood, Wei Shern Lee, Greta Gillies, Kate Pope, Michael Wilson, Paul J Lockhart, Alexander Dobrovic, Ingrid E Scheffer, Melanie Bahlo, Richard J Leventer, Ryan Lister, Samuel F Berkovic, Michael S Hildebrand

**Affiliations:** 1 Department of Medicine (Austin Health), University of Melbourne, Melbourne, Victoria 3084, Australia; 2 Brain and Mind Centre, Sydney Medical School, The University of Sydney, Sydney, New South Wales 2050, Australia; 3 Australian Research Council Centre of Excellence in Plant Energy Biology, School of Molecular Sciences, The University of Western Australia, Perth, Western Australia 6009, Australia; 4 Harry Perkins Institute of Medical Research, Perth, Western Australia 6150, Australia; 5 Murdoch Children’s Research Institute, Melbourne, Victoria 3052, Australia; 6 Department of Paediatrics, University of Melbourne, Royal Children’s Hospital, Melbourne, Victoria 3052, Australia; 7 Department of Neurology, Royal Children’s Hospital, Melbourne, Victoria 3052, Australia; 8 Department of Neurosciences, Queensland Children’s Hospital, Brisbane, Queensland 4101, Australia; 9 Department of Anatomical Pathology, St. Vincent’s Hospital, Melbourne, Victoria 3065, Australia; 10 School of Cancer Medicine, La Trobe University, Melbourne, Victoria 3086, Australia; 11 Department of Clinical Pathology, University of Melbourne, Melbourne, Victoria 3010, Australia; 12 Department of Neurosurgery, Royal Children’s Hospital, Melbourne, Victoria 3052, Australia; 13 Neurosurgical Department, Queensland Children’s Hospital, Brisbane, Queensland 4101, Australia; 14 Translational Genomics and Epigenomics Laboratory, Olivia Newton-John Cancer Research Institute, Melbourne, Victoria 3084, Australia; 15 Population Health and Immunity Division, The Walter and Eliza Hall Institute of Medical Research, Melbourne, Victoria 3052, Australia; 16 Department of Medical Biology, University of Melbourne, Melbourne, Victoria 3010, Australia

**Keywords:** cell-free DNA, cerebrospinal fluid, focal epilepsy, liquid biopsy, somatic mutations

## Abstract

Brain somatic mutations are an increasingly recognized cause of epilepsy, brain malformations and autism spectrum disorders and may be a hidden cause of other neurodevelopmental and neurodegenerative disorders. At present, brain mosaicism can be detected only in the rare situations of autopsy or brain biopsy. Liquid biopsy using cell-free DNA derived from cerebrospinal fluid has detected somatic mutations in malignant brain tumours. Here, we asked if cerebrospinal fluid liquid biopsy can be used to detect somatic mosaicism in non-malignant brain diseases. First, we reliably quantified cerebrospinal fluid cell-free DNA in 28 patients with focal epilepsy and 28 controls using droplet digital PCR. Then, in three patients we identified somatic mutations in cerebrospinal fluid: in one patient with subcortical band heterotopia the *LIS1* p. Lys64* variant at 9.4% frequency; in a second patient with focal cortical dysplasia the *TSC1* p. Phe581His*6 variant at 7.8% frequency; and in a third patient with ganglioglioma the *BRAF* p. Val600Glu variant at 3.2% frequency. To determine if cerebrospinal fluid cell-free DNA was brain-derived, whole-genome bisulphite sequencing was performed and brain-specific DNA methylation patterns were found to be significantly enriched (*P* = 0.03). Our proof of principle study shows that cerebrospinal fluid liquid biopsy is valuable in investigating mosaic neurological disorders where brain tissue is unavailable.

## Teaser

Somatic mutations can cause brain diseases. Currently, the only established route to study the brain-only somatic mutations is by sequencing brain tissues obtained from neurosurgery or autopsy. Ye *et al.* report a new strategy to use CSF cell-free DNA to detect brain-only somatic mutations as a surrogate for brain tissues.

## Graphical Abstract

**Graphical Abstract:**
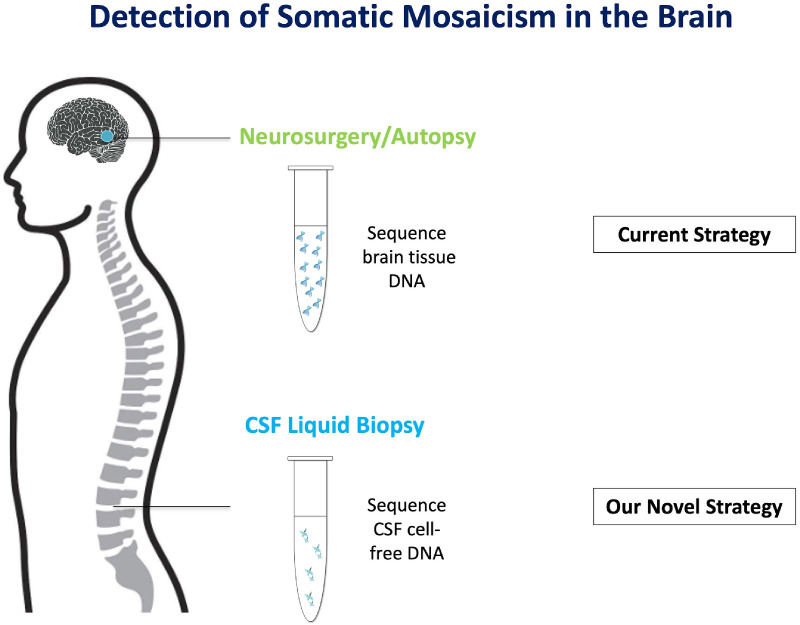

## Introduction

Cell-free DNA is externalized, fragmented DNA found in bodily fluids and is the product of programmed cell death. Fragments are predominantly short with a predominant peak at ∼167 base pairs (bp) (Chandrananda *et al.*, 2015). Analysis of cell-free DNA, typically from peripheral blood, is now clinically used as a liquid biopsy for diagnosis and monitoring of cancer (Crowley *et al.*, 2013), obstetric practice (Bianchi and Chiu, 2018) and as a biomarker for allograft rejection after organ transplantation (Goh *et al.*, 2017). Interrogation of CSF-derived cell-free DNA allows detection of mosaic somatic mutations in malignant brain tumours (Pan *et al.*, 2015; Wang *et al.*, 2015; Garcia-Romero *et al.*, 2019), but its application to diagnosis of non-malignant brain diseases has not been explored.

While a significant proportion of patients with neurological and neurodevelopmental disorders have disease-causing germline mutations (Allen *et al.*, 2013; Iossifov *et al.*, 2014), many remain unsolved after routine genetic testing. Recent studies indicate that some of these unsolved cases may be explained by somatic mutation (Thomas and Berkovic, 2014). Mosaic somatic mutations have been identified in patients with neurological disorders including brain malformations and epilepsy (Poduri *et al.*, 2012; D’Gama *et al.*, 2017; Winawer *et al.*, 2018; Sim *et al.*, 2019; Ye *et al.*, 2019) and autism spectrum disorder (D’Gama *et al.*, 2015). Somatic mutations occur post-zygotically and, if confined to brain, are difficult or impossible to detect by conventional sequence analysis of lymphocyte-derived DNA (Poduri *et al.*, 2013). At present, the only established route to discovery of somatic mutations in brain is via the privileged situation of having brain tissue from surgical or autopsy specimens; this is not generally applicable to common epilepsies and other neurological and neurodevelopmental disorders. An alternative route is needed to detect the brain-only somatic mutations when brain tissue is not available. Here, we provide proof of principle that CSF liquid biopsies can be used as a surrogate to detect somatic mosaicism, without the need for brain tissue.

## Materials and methods

### Study approval

The Human Research Ethics Committees of The Royal Children’s Hospital (Project No. 29077F) and Austin Health, Melbourne, Australia (Project No. H2007/02961) approved this study. Written informed consent was obtained from all participants or their parents or legal guardians according to the Declaration of Helsinki.

### Clinical cohorts

For initial CSF cell-free DNA quantitation, we studied 28 patients with epilepsy (mean age, 7.8 ± 5 years old) and 28 controls without epilepsy (mean age, 46.9 ± 15.6 years old). They were recruited through Austin Health, Royal Children’s Hospital, and Queensland Children’s Hospital, Australia. All patients had drug-resistant, lesional, focal epilepsy requiring surgical treatment (Supplementary Table 1). Controls had been referred for non-epilepsy presentations (e.g. persistent headache). CSF was collected in Cell-Free DNA BCT^®^ (Strek, La Vista, NE) tubes via dural puncture immediately after craniotomy (epilepsy patients) or lumbar puncture (controls). Mean CSF volume collected was 2 ml (range, 0.25–10.5 ml).

For molecular diagnosis, CSF samples were obtained by lumbar puncture (patient 1, separate from the 28 patients used for the quantitation) and peri-operative dural puncture (patients 2 and 3, from the cohort of 28 patients used for quantitation) in patients with known or suspected brain mosaic mutations.

### DNA extraction

Cell-free DNA was extracted from CSF using the QIAamp Circulating Nucleic Acid Kit (Qiagen, Hilden, Germany): CSF was centrifuged at 1000 × *g* at 4°C for 10 min then extracted according to the manufacturer’s protocol. For blood and brain, genomic DNA was extracted using standard methods (Qiagen QIAamp DNA Maxi Kit or AllPrep DNA/RNA Kit, Hilden, Germany).

### Droplet digital PCR

For quantitation of total cell-free DNA in CSF, we used a PrimerPCR™ Mutation Assay (Catalog: 10049047; Bio-Rad, Hercules, CA) that we previously optimized in-house (Hildebrand *et al.*, 2018) to detect the *GNAQ* c.548G wild-type allele (NM_002072.5:c.548G) as a reference for cell-free DNA copies (Supplementary Table 2). Total cell-free DNA copies and cell-free DNA concentration (copies/ml CSF and ng/ml CSF) were calculated using reported formulas (Supplementary Methods).

For molecular diagnosis, we used the in-house *LIS1* NM_000430.4:c.190A>T (p.Lys64*) ddPCR assay (Damiano *et al.*, 2017), a customized *TSC1* NM_000368.5:c.1741_1742delTT (p.Phe581His*6) ddPCR assay (Thermo Fisher Scientific, Waltham, MA), or a PrimerPCR™ *BRAF* NM_004333.6:c.1799T>A (p.Val600Glu) assay (ID: dHsaCP2000027 and dHsaCP2000028; Bio-Rad, Hercules, CA) (Supplementary Table 2). Droplet generation, PCR cycling and droplet reading were performed on the QX200 ddPCR system according to the manufacturer’s recommendations (Bio-Rad). Limit of detection tests for each assay were conducted using Gblock synthetic DNA fragments (Supplementary Methods). The limit of detection for all three assays was 0.25% variant allele frequency (VAF) (Supplementary Figs 1–3).

### Whole genome bisulphite sequencing and cell-of-origin analysis of cell-free DNA

To determine origin of CSF cell-free DNA, we examined cell-free DNA for brain-specific DNA methylation patterns. We analysed four control CSF samples and patient 1’s CSF sample by whole-genome bisulphite sequencing (WGBS) followed by methylK analysis (Supplementary Methods). Sequencing libraries were prepared using Accel-NGS Methyl-Seq DNA Library Kit (Swift Biosciences, USA) according to the manufacturer’s instructions for low-input methylomes. Pronex beads were used to deplete adapter dimers and enrich library fragments prior to sequencing. We sequenced 2–5 million paired-end reads for each CSF sample. All samples had 0.024-0.051x coverage for cytosines in the CG dinucleotide sequence context and 0.025–0.055x coverage for cytosines in the CH dinucleotide sequence context. In addition, we acquired five WGBS data sets from Swift Biosciences that were produced from peripheral blood plasma cell-free DNA in which the libraries were made with the same library preparation kit and sequenced to a comparable depth to CSF samples.

To identify the cell-of-origin of CSF cell-free DNA, we established an analytical method for assigning single WGBS reads. Briefly, publicly available WGBS from 11 cell types-of-interest [e.g. blood Cells (ENCODE) and central nervous system (CNS) cell types (Lister *et al.*, 2013)] provided reference cell-type data sets from which DNA methylation fractional measurements were converted into cell-type specific reference sequences (FASTA). Sub-sequences (k-mers) were indexed from all cell-type references (*n* = 11) using Kallisto software (Bray *et al.*, 2016). Trimmed and truncated WGBS reads from CSF (*n* = 5) and plasma (*n* = 5) cell-free DNA were assigned (pseudo-aligned) to their cell-of-origin by hash table lookup using Kallisto software (Bray *et al.*, 2016) and only high-quality reads assigned to DNA methylation reference sequences of a unique cell-type were counted and used for statistical analysis (Supplementary Methods, Supplementary Fig. 4).

### Statistical analysis

Student’s *t*-tests were used to compare counts of WGBS reads assigned to CNS cells and blood cells between CSF cell-free DNA and plasma cell-free DNA. All statistical tests were performed using SPSS Statistics (SPSS 25, SPSS Inc., Chicago, IL, USA). A *P*-value of <0.05 was considered statistically significant.

### Data availability

The authors confirm that the data supporting the findings of this study are available within the article and its Supplementary Material. Raw data of this study are available from the corresponding authors, upon reasonable request.

## Results

First, to establish that CSF cell-free DNA can be reliably quantified, we studied 28 patients with focal epilepsy and 28 controls. We quantified cell-free DNA in CSF samples by interrogating the *GNAQ* c.548G wild-type allele as a reference for cell-free DNA copies using ddPCR. Cell-free DNA copies were successfully detected and reliably quantified in all patient CSF samples (examples of ddPCR output are shown in Supplementary Fig. 5). The median value of cell-free DNA concentration was 502 copies/ml CSF (∼1.5 ng/ml CSF) in paediatric patients with epilepsy and 61 copies/ml CSF (∼0.18 ng/ml CSF) in adult controls, respectively (Supplementary Table 3 and Fig. 6). This demonstrated that cell-free DNA could be reliably detected in CSF from subjects with epilepsy and controls.

Next, to establish molecular diagnosis, we investigated CSF cell-free DNA from three patients. Two patients had a known mosaic pathogenic variant in brain tissue which was also found in CSF cell-free DNA. In the third patient, the mutation was first identified by CSF cell-free DNA analysis and later confirmed in brain tissue.

**Figure 1:**
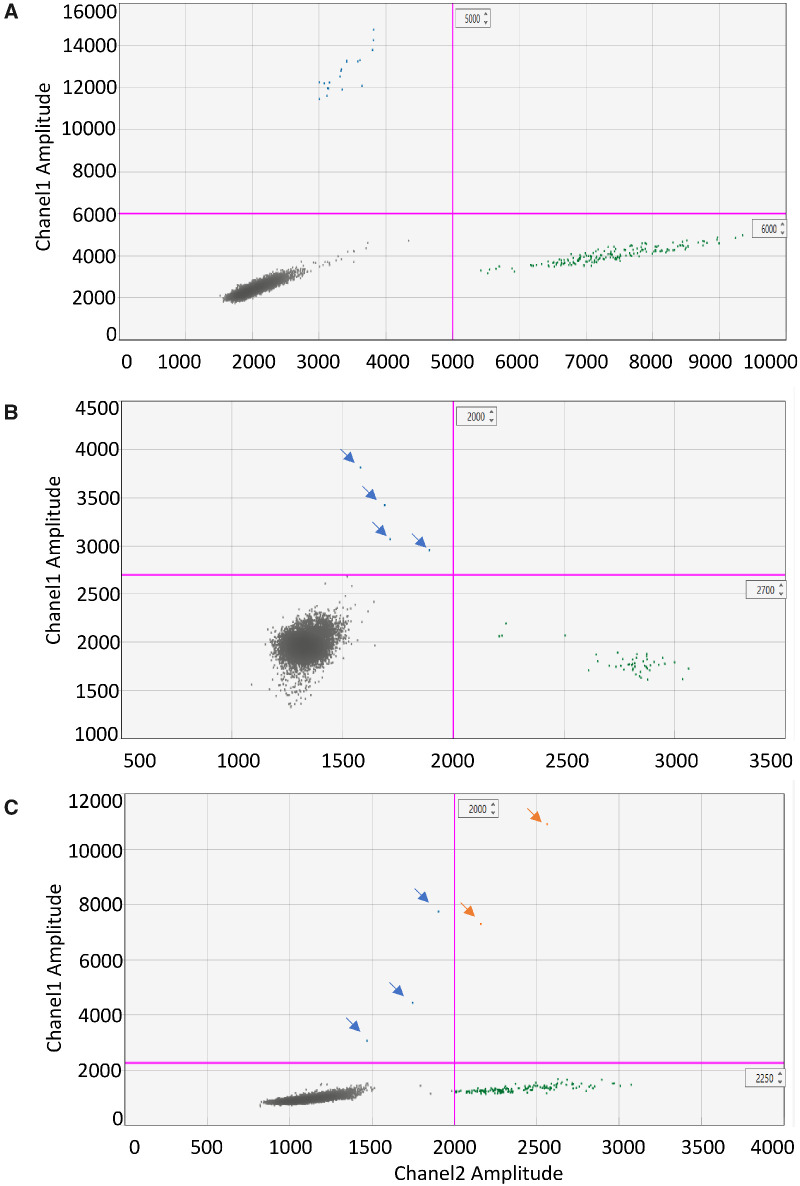
**ddPCR 2D-plot for molecular diagnosis in CSF from three patients with lesional focal epilepsy.** (**A**). 2D-Plot showing droplets positive (blue, upper left quadrant) for *LIS1* Lys64* in patient 1. CSF cell-free DNA was tested twice with similar VAF, then data were combined. Mutant concentration 3.61 copies/μl; wild-type concentration 35 copies/μl; VAF: 9.4%. (B) 2D-plot showing droplets positive for *TSC1* p. Phe581His*6 in patient 2. CSF cell-free DNA: mutant concentration 0.47 copies/μl; wild-type concentration 5.58 copies/μl; VAF: 7.8%. (**C**) 2D-plot showing droplets positive for *BRAF* Val600Glu in patient 3. CSF cell-free DNA: mutant concentration 0.42 copies/μl; wild-type concentration 12.7 copies/μl; VAF: 3.2%. Green droplets are wild-type copies, orange droplets are double-positive copies and grey droplets are empty. VAF: variant allele frequency.

Patient 1 was a 41-year-old woman with drug-resistant focal epilepsy, mild intellectual disability and bilateral posterior subcortical band heterotopia. She had the somatic mosaic *LIS1* mutation c.190A*>*T (p.Lys64*) in blood (13% VAF) (Jamuar *et al.*, 2014), saliva (∼13% VAF) and a 25-year-old formalin-fixed paraffin-embedded brain tissue (∼5% VAF) (Damiano *et al.*, 2017). We obtained 6 ml of CSF via lumbar puncture and extracted cell-free DNA (444 copies/ml, ∼1.3 ng/ml). The *LIS1* p. Lys64* mutation was detected in CSF cell-free DNA on two independent tests with a mean VAF of 9.4% (Table 1, Fig. 1A, Supplementary Table 4 and Fig. 7A).

**Table 1:** Genetic diagnosis of patients with epilepsy and brain malformations

	Clinical diagnosis	CSF volume (ml)	cfDNA concentration (copies/ml CSF)	Gene mutation	**Brain VAF (%)** ^a^	**CSF VAF (%)** ^a^	**Blood VAF** ^a^
**Patient 1**	Bilateral posterior subcortical band heterotopia	6	444	*LIS1* c.190A>T (p.Lys64^a^)	5.8^b^	9.4	14.5%
**Patient 2**	Left inferior temporal gyrus focal cortical dysplasia IIb	1.5	789	*TSC1* c.1741_1742delTT (p.Phe581His^a^6)	2.3	7.8	Undetectable
**Patient 3**	Left medial temporal ganglioglioma	3	1058	*BRAF* c.1799T>A (p.Val600Glu)	20.4	3.2	Undetectable

Abbreviation: VAF, variant allele frequency.

a Measured by ddPCR.

b Damiano *et al.* (2017).

Patient 2 was a 3-year-old girl with a solitary, left inferior temporal gyrus focal cortical dysplasia type IIB, but no other clinical or imaging features of tuberous sclerosis. She had the brain-only somatic *TSC1* mutation c.1741_1742delTT (p.Phe581His*6) detected at 2.8% VAF by deep sequencing in the resected lesion. We obtained 1.5 ml of CSF via dural puncture prior to resective surgery and isolated cell-free DNA (789 copies/ml). The *TSC1* p*.* Phe581His*6 mutation was detected at 7.8% VAF in CSF; using the same ddPCR assay, we re-confirmed this mutation in brain tissue (2.3% VAF), and it was undetectable in blood (Table 1, Fig. 1B and Supplementary Table 4 and Fig. 7B).

Patient 3 was a 2-year-old boy with a left medial temporal ganglioglioma who had received no molecular testing. We obtained 3 ml of CSF via dural puncture prior to resective surgery and isolated cell-free DNA (1058 copies/ml). Since 50–60% of patients with ganglioglioma have a brain-only recurrent somatic *BRAF* mutation c.1799T>A (p.Val600Glu) (Koelsche *et al.*, 2013; Berghoff and Preusser, 2014), we screened for this mutation using a targeted ddPCR assay. We detected this mutation at 3.2% VAF in CSF cell-free DNA; subsequent testing confirmed that the mutation was present at 20.4% VAF in his ganglioglioma, and undetectable in his blood (Table 1, Fig. 1C and Supplementary Table 4 and Fig. 7C).

**Figure 2:**
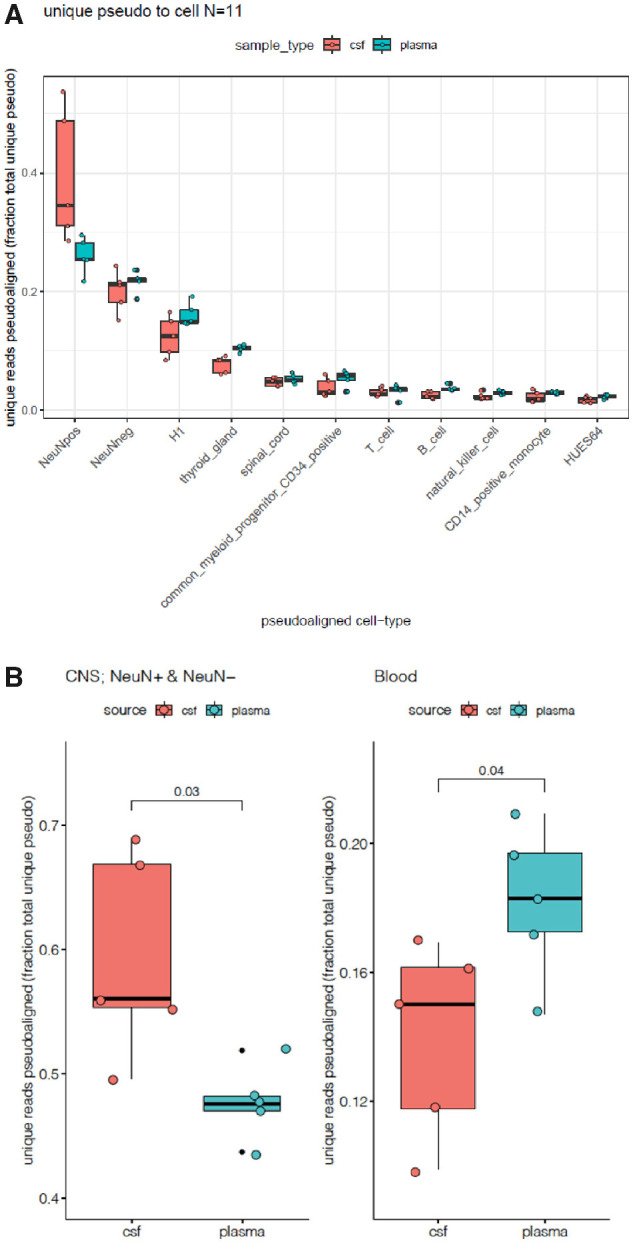
**Enrichment of CNS-derived cell-free DNA in CSF samples.** (**A**) Unique pseudo-alignment of cell-free DNA from CSF and plasma to reference sequences/k-mers from 11 tissues. (**B**) Unique sequence pseudo-alignments of cell-free DNA from CSF and plasma to brain and blood cells. CSF-derived cell-free DNA has significantly higher number of unique reads pseudo-aligned to brain compared to plasma-derived cell-free DNA (*P*** **=** **0.03), whereas plasma-derived cell-free DNA was significantly enriched for unique reads pseudo-aligned to blood cells (*P*** **=** **0.04).

Finally, to confirm that cell-free DNA in CSF is brain-derived, we examined cell-free DNA for brain-specific DNA methylation patterns. We showed for CSF cell-free DNA that a greater proportion of WGBS reads pseudo-aligned to brain cells than blood cells (Fig. 2A and Supplementary Fig. 4). We assessed brain cell-derived (NeuN+ neurons, NeuN-glia) and blood cell-derived (CD34+ myeloid progenitors, T-cells, B-cells, Natural Killer cells and CD14+ monocytes) contributions to cell-free DNA within CSF and plasma. We observed that CSF-derived cell-free DNA was significantly (*P* = 0.03) enriched for unique reads pseudo-aligned to brain cells, whereas plasma-derived cell-free DNA had a significantly (*P* = 0.04) higher number of unique reads pseudo-aligned to blood cells (Fig. 2B). The small fraction of NeuN k-mer within plasma likely reflects a lack of specificity in established methods for deconvolution of CpH methylation in the pseudo-aligned reads from blood genomes. Our findings indicate that the cell-free DNA in CSF is largely brain derived.

## Discussion

Somatic mutations have been well studied in malignant tumours, but their role in neurological and neurodevelopmental disorders remains poorly understood. With the improving throughput and depth of genetic screening technologies, the contribution of somatic mutations is increasingly being recognized.

There are many unsolved brain diseases for which a genetic aetiology has been invoked. In epilepsy, however, only a minority of patients have a known molecular cause. By study of privileged brain tissue samples, usually from epilepsy surgery, some cases, including but not limited to brain malformations, are now known to be due to somatic mutation in the brain (Poduri *et al.*, 2012; D’Gama *et al.*, 2015, 2017; D’Gama and Walsh, 2018; Winawer *et al.*, 2018). The discovery of these mutations in post-mortem tissue in individuals with autism spectrum disorder (D’Gama *et al.*, 2015) and Alzheimer’s disease (Park *et al.*, 2019) implicates this mechanism more broadly in neurodevelopmental (D’Gama and Walsh, 2018) and neurodegenerative disorders (Lodato and Walsh, 2019). It has even been repeatedly hypothesized that somatic mutations contribute to the pathogenesis of Parkinson disease (Engeholm and Gasser, 2013; Proukakis *et al.*, 2013). Analysing brain tissue in living patients with autism spectrum disorder, Alzheimer’s disease or Parkinson disease is not feasible as these patients do not have surgery (except deep-brain stimulation in rare cases where tissue is not resected) as part of their clinical treatment.

Our results show that there is enough cell-free DNA in CSF to make a targeted liquid biopsy possible. We found cell-free DNA concentration is higher in the patients with epilepsy compared to the controls. This might be because seizures can trigger an increase in apoptosis of brain cells, leading to shedding of more cell-free DNA into CSF. But there are multiple confounding factors that may contribute to the difference: (i) age: the patients were children while the controls were adults and apoptosis is elevated in the brain during development; (ii) CSF collection: patient CSF was collected via dural puncture whereas control CSF was collected via lumbar puncture. It would have been ideal to obtain CSF via dural puncture from some controls, but we did not have institutional ethics approval for this.

Our findings suggest that CSF liquid biopsy is a viable surrogate for brain tissue to establish molecular diagnosis of brain somatic mosaicism, with implications for clinical care. These findings may provide new insights into the molecular genetic factors behind epilepsy and brain malformations, as well as other neurodevelopmental and neurodegenerative diseases potentially linked to brain somatic mutations where neurosurgery is not standard of care.

There are three main challenges to clinical implementation of CSF liquid biopsy for diagnostic purposes. First, the technical issue of obtaining high-quality DNA from CSF. We have shown that the level of CNS-derived cell-free DNA in the CSF of epilepsy patients is sufficient to enable targeted molecular interrogation of somatic mutations. Second, the biological issue of detection of low concentrations of mutant DNA in CSF, especially if the mutation is presumed localized to a small lesion; detection of mutations in patients 2 and 3 shows that this challenge may be overcome. Third, we performed only targeted mutational analysis in three patients to establish the proof of principle of this approach. The remaining patients were not tested due to lack of a candidate variant from tissue analysis for targeted assays. In the future, unbiased, high depth screening, with optimized low-input genomic capture for CSF cfDNA, will be required to identify a novel somatic mutation genome-wide in establishing broader clinical utility.

## Conclusion

As precision treatments for neurogenetic disorders are being investigated, such as mTOR inhibitors for cortical dysplasia, implementing CSF liquid biopsy for aetiological diagnosis will become increasingly important as a surrogate for brain-derived DNA to diagnose brain-specific somatic disorders.

## Supplementary material

Supplementary material is available at *Brain Communications* online.

## Supplementary Material

fcaa235_Supplementary_Data

## Abbreviations

CNS =: central nervous system
CSF =: cerebrospinal fluid
ddPCR =: droplet digital PCR
VAF =: variant allele frequency
WGBS =: whole-genome bisulphite sequencing

## References

[fcaa235-B1] Allen AS , Berkovic SF , Cossette P , Delanty N , Dlugos D , Eichler EE , et al De novo mutations in epileptic encephalopathies. Nature 2013; 501: 217–21. 23934111 10.1038/nature12439 PMC3773011

[fcaa235-B2] Berghoff AS , Preusser M. BRAF alterations in brain tumours: molecular pathology and therapeutic opportunities. Curr Opin Neurol 2014; 27: 689–96. 25268071 10.1097/WCO.0000000000000146

[fcaa235-B3] Bianchi DW , Chiu RWK. Sequencing of circulating cell-free DNA during pregnancy. N Engl J Med 2018; 379: 464–73. 30067923 10.1056/NEJMra1705345 PMC10123508

[fcaa235-B4] Bray NL , Pimentel H , Melsted P , Pachter L. Near-optimal probabilistic RNA-seq quantification. Nat Biotechnol 2016; 34: 525–7. 27043002 10.1038/nbt.3519

[fcaa235-B5] Chandrananda D , Thorne NP , Bahlo M. High-resolution characterization of sequence signatures due to non-random cleavage of cell-free DNA. BMC Med Genomics 2015; 8: 29. 26081108 10.1186/s12920-015-0107-z PMC4469119

[fcaa235-B6] Crowley E , Di Nicolantonio F , Loupakis F , Bardelli A. Liquid biopsy: monitoring cancer-genetics in the blood. Nat Rev Clin Oncol 2013; 10: 472–84. 23836314 10.1038/nrclinonc.2013.110

[fcaa235-B7] Damiano JA , Do H , Ozturk E , Burgess R , Kalnins R , Jones NC , et al Sensitive quantitative detection of somatic mosaic mutation in “double cortex” syndrome. Epileptic Disord 2017; 19: 450–5. 29258966 10.1684/epd.2017.0944

[fcaa235-B8] D’Gama AM , Pochareddy S , Li M , Jamuar SS , Reiff RE , Lam A-TN , et al Targeted DNA sequencing from autism spectrum disorder brains implicates multiple genetic mechanisms. Neuron 2015; 88: 910–7. 26637798 10.1016/j.neuron.2015.11.009 PMC4672379

[fcaa235-B9] D’Gama AM , Walsh CA. Somatic mosaicism and neurodevelopmental disease. Nat Neurosci 2018; 21: 1504–14. 30349109 10.1038/s41593-018-0257-3

[fcaa235-B10] D’Gama AM , Woodworth MB , Hossain AA , Bizzotto S , Hatem NE , LaCoursiere CM , et al Somatic mutations activating the mTOR pathway in dorsal telencephalic progenitors cause a continuum of cortical dysplasias. Cell Reports 2017; 21: 3754–66. 29281825 10.1016/j.celrep.2017.11.106 PMC5752134

[fcaa235-B11] Engeholm M , Gasser T. Parkinson’s disease: is it all in the genes? Mov Disord 2013; 28: 1027–9. 23868561 10.1002/mds.25611

[fcaa235-B12] Garcia-Romero N , Carrion-Navarro J , Areal-Hidalgo P , Ortiz de Mendivil A , Asensi-Puig A , Madurga R , et al BRAF V600E detection in liquid biopsies from pediatric central nervous system tumors. Cancers 2019; 12: 66. 10.3390/cancers12010066 PMC7016762 31881643

[fcaa235-B13] Goh SK , Muralidharan V , Christophi C , Do H , Dobrovic A. Probe-free digital PCR quantitative methodology to measure donor-specific cell-free DNA after solid-organ transplantation. Clin Chem 2017; 63: 742–50. 28100495 10.1373/clinchem.2016.264838

[fcaa235-B14] Hildebrand MS , Harvey AS , Malone S , Damiano JA , Do H , Ye Z , et al Somatic GNAQ mutation in the forme fruste of Sturge-Weber syndrome. Neurol Genet 2018; 4: e236. 29725622 10.1212/NXG.0000000000000236 PMC5931068

[fcaa235-B15] Iossifov I , O ssif BJ , Sanders SJ , Ronemus M , Krumm N , Levy D , et al The contribution of de novo coding mutations to autism spectrum disorder. Nature 2014; 515: 216–21. 25363768 10.1038/nature13908 PMC4313871

[fcaa235-B16] Jamuar SS , Lam A-TN , Kircher M , D rche AM , Wang J , Barry BJ , et al Somatic mutations in cerebral cortical malformations. N Engl J Med 2014; 371: 733–43. 25140959 10.1056/NEJMoa1314432 PMC4274952

[fcaa235-B17] Koelsche C , Wohrer A , Jeibmann A , Schittenhelm J , Schindler G , Preusser M , et al Mutant BRAF V600E protein in ganglioglioma is predominantly expressed by neuronal tumor cells. Acta Neuropathol 2013; 125: 891–900. 23435618 10.1007/s00401-013-1100-2

[fcaa235-B18] Lister R , Mukamel EA , Nery JR , Urich M , Puddifoot CA , Johnson ND , et al Global epigenomic reconfiguration during mammalian brain development. Science 2013; 341: 1237905. 23828890 10.1126/science.1237905 PMC3785061

[fcaa235-B19] Lodato MA , Walsh CA. Genome aging: somatic mutation in the brain links age-related decline with disease and nominates pathogenic mechanisms. Hum Mol Genet 2019; 28: R197–206. 31578549 10.1093/hmg/ddz191 PMC6872434

[fcaa235-B20] Pan W , Gu W , Nagpal S , Gephart MH , Quake SR. Brain tumor mutations detected in cerebral spinal fluid. Clin Chem 2015; 61: 514–22. 25605683 10.1373/clinchem.2014.235457 PMC5412506

[fcaa235-B21] Park JS , Lee J , Jung ES , Kim MH , Kim IB , Son H , et al Brain somatic mutations observed in Alzheimer’s disease associated with aging and dysregulation of tau phosphorylation. Nat Commun 2019; 10: 3090. 31300647 10.1038/s41467-019-11000-7 PMC6626023

[fcaa235-B22] Poduri A , Evrony GD , Cai X , Elhosary PC , Beroukhim R , Lehtinen MK , et al Somatic activation of AKT3 causes hemispheric developmental brain malformations. Neuron 2012; 74: 41–8. 22500628 10.1016/j.neuron.2012.03.010 PMC3460551

[fcaa235-B23] Poduri A , Evrony GD , Cai X , Walsh CA. Somatic mutation, genomic variation, and neurological disease. Science 2013; 341: 1237758. 23828942 10.1126/science.1237758 PMC3909954

[fcaa235-B24] Proukakis C , Houlden H , Schapira AH. Somatic alpha-synuclein mutations in Parkinson’s disease: hypothesis and preliminary data. Mov Disord 2013; 28: 705–12. 23674490 10.1002/mds.25502 PMC3739940

[fcaa235-B25] Sim NS , Ko A , Kim WK , Kim SH , Kim JS , Shim KW , et al Precise detection of low-level somatic mutation in resected epilepsy brain tissue. Acta Neuropathol 2019; 138: 901–12. 31377847 10.1007/s00401-019-02052-6

[fcaa235-B26] Thomas RH , Berkovic SF. The hidden genetics of epilepsy-a clinically important new paradigm. Nat Rev Neurol 2014; 10: 283–92. 24733163 10.1038/nrneurol.2014.62

[fcaa235-B27] Wang Y , Springer S , Zhang M , McMahon KW , Kinde I , Dobbyn L , et al Detection of tumor-derived DNA in cerebrospinal fluid of patients with primary tumors of the brain and spinal cord. Proc Natl Acad Sci USA 2015; 112: 9704–9. 26195750 10.1073/pnas.1511694112 PMC4534284

[fcaa235-B28] Winawer MR , Griffin NG , Samanamud J , Baugh EH , Rathakrishnan D , Ramalingam S , et al Somatic SLC35A2 variants in the brain are associated with intractable neocortical epilepsy. Ann Neurol 2018; 83: 1133–46. 29679388 10.1002/ana.25243 PMC6105543

[fcaa235-B29] Ye Z , McQuillan L , Poduri A , Green TE , Matsumoto N , Mefford HC , et al Somatic mutation: the hidden genetics of brain malformations and focal epilepsies. Epilepsy Res 2019; 155: 106161. 31295639 10.1016/j.eplepsyres.2019.106161

