# Corrigendum to: Cerebrospinal fluid liquid biopsy for detecting somatic mosaicism in brain

**DOI:** 10.1093/braincomms/fcab103

**Published:** 2021-06-17

Zimeng Ye, Zac Chatterton, Jahnvi Pflueger, John A Damiano, Lara McQuillan, A. Simon Harvey, Stephen Malone, Hongdo Do, Wirginia Maixner, Amy Schneider, Bernadette Nolan, Martin Wood, Wei Shern Lee, Greta Gillies, Kate Pope, Michael Wilson, Paul J Lockhart, Alexander Dobrovic, Ingrid E Scheffer, Melanie Bahlo, Richard J Leventer, Ryan Lister, Samuel F Berkovic, Michael S Hildebrand. Cerebrospinal fluid liquid biopsy for detecting somatic mosaicism in brain. Brain Communications 2021. doi:10.1093/braincomms/fcaa235.

In the originally published version of this manuscript, there was an error in the spelling of Co-author A. Simon Harvey’s name. The full name should read: “A. Simon Harvey” instead of “Anthony Simon Harvey”. This has now been corrected online.

