# Supplementary material for: Cerebrospinal fluid liquid biopsy for detecting somatic mosaicism in brain
Source: Brain Commun. 2021 Jan 21;3(1):fcaa235. doi: 10.1093/braincomms/fcaa235 (PMC7954394; doi:10.1093/braincomms/fcaa235)
Supplement: fcaa235_Supplementary_Data [file fcaa235_Supplementary_Data.docx]

**Supplementary Appendix**

This appendix has been provided by the authors to give readers additional information about their work.

Supplement to: Ye, Chatterton, Pflueger, et al. Cerebrospinal fluid liquid biopsy for detecting somatic mosaicism in brain

**Table of Contents**

Methods……………………………………………………………………………………. 3-8

Supplementary Figure 1. Limit of Detection for *LIS1* p.Lys64* ddPCR Assay……….……..9

Supplementary Figure 2. Limit of Detection for *TSC1* p.Phe581His*6 ddPCR Assay……..10

Supplementary Figure 3. Limit of Detection for *BRAF* p. Val600Glu ddPCR Assay……....11

Supplementary Figure 4. Pseudoalignment of WGBS cell-free DNA using DNA Methylation K-mers….................................................................................................................................12

Supplementary Figure 5. Examples of ddPCR output for CSF cell-free DNA quantitation...13

Supplementary Figure 6. CSF cfDNA Concentration in Epilepsy Patients and Controls.…..14

Supplementary Figure 7. ddPCR 2D-plot for Brain and Blood in 3 Patients with Lesional Focal Epilepsy …………………………………………………………………………….…15

Supplementary Table 1. Clinical and Genetic Information on the Patients with Epilepsy…..16

Supplementary Table 2. Primer and Probe Sequences and Annealing Temperatures for ddPCR Assays .………………………………………………………………………………17

Supplementary Table 3. CSF cell-free DNA Concentration in Epilepsy Patients and Controls ……………………………………………………………………………………...18

Supplementary Table 4. ddPCR Raw Data of the 3 Patients with Focal Epilepsy and Brain Malformations ……………………………………………………………………………….19

Additional References . . . . . . . . . . . . . . . . . . . . . . . . . . . . . . . . . . . . . . . . . . . . . . . . . . . . . . .20

**METHODS**

**1. Droplet digital PCR (ddPCR)**

Probes and primers (**Supplementary Table 2**) were mixed with 2x ddPCR Supermix for probe (Bio-Rad) at 250 nM and 900 nM final concentrations for each probe and each of the primers, respectively, and mixed with 10 μl of neat cell-free DNA sample to a final volume of 23 μl. Positive controls, no mutation controls and no template controls were used for each test. Twenty microliters of reactions were loaded in an eight-channel droplet generator cartridge (Bio-Rad) and droplets were generated with 70 μl of droplet generation oil (Bio-Rad) by using the manual QX200 Droplet Generator. Following droplet generation samples were manually transferred to a 96-well PCR plate, heat-sealed and amplified on a C1000 Touch thermal cycler using the following cycling conditions: 95°C for 10 minutes for one cycle, followed by 40 cycles at 94°C for 30 seconds and Tm for 60 seconds, one cycle at 98°C for 10 minutes and 12°C for infinite. Post-PCR products were read on the QX200 droplet reader (Bio-Rad) and analysed using the QuantaSoft software.

**2. cell-free DNA Quantitation Calculation**

For quantitation of cell-free DNA in CSF we used a commercially available PrimerPCR^TM^ Mutation Assay (ID: 10049047; Bio-Rad, Hercules, CA) to detect the *GNAQ* c.548G wild-type allele as a reference for cell-free DNA level. Total copies and concentration of cell-free DNA were calculated using the following formula:

*GNAQ* WT copies from QuantaSoft software = 𝑥 (copies/μl reaction mixture)

Extraction elution volume = 𝑉e (μl)

CSF sample volume = 𝑉c (mL)

$CSF cell-free DNA concentration=\frac{\frac{23*x}{10}*Ve}{Vc}$ (copies/mL CSF)

Estimated CSF cell-free DNA concentration by ng/mL $\approx$0.003 x $\frac{\frac{23*x}{10}*Ve}{Vc}$ (ng/mL CSF)

The equation uses the *GNAQ* WT copies in the reaction mixture (calculated by QuantaSoft software) and multiplies it by the reaction mixture volume (23 μL) to calculate the total *GNAQ* WT copies in the reaction mixture. This value is divided by the 10 μL volume of cell-free DNA extraction elution that was used in the reaction mixture to obtain the *GNAQ* WT copies in the cell-free DNA elution. This elution concentration is then multiplied by the total elution volume to give the total *GNAQ* WT copies in the CSF sample, which is then divided by the starting CSF sample volume (between 0.25 and 10.5 mL) to give the final CSF cell-free DNA concentration. The approximate concentration in ng/mL CSF was calculated based on the mass of 1 haploid human genome is 0.003 ng (Pan *et al*, 2015).

**3. Limit of Detection Tests for Each ddPCR Assay**

Mutant Gblocks and wild-type Gblocks for *LIS1* Lys64*, *TSC1* Phe581His*6 and *BRAF* Val600Glu were purchased from Integrated DNA Technologies (IDT, Iowa City, IA). Limit of detection of each ddPCR assay was established by serially diluting mutant Gblocks into paired wild-type Gblocks in order to obtain different mutant/(mutant + wild-type) ratios: 2.5, 1, 0.5, 0.25, 0.1, 0.05 and 0.01%. Gblocks were used at low concentration to mimic the low concentration of CSF cell-free DNA samples. Three or more positive droplets were required to achieve true positive calls (Uchiyama et al, 2016; Rowlands et al, 2019). Droplet generation, PCR cycling and droplet reading were performed with the protocol described above.

**4. Methylation Study for cell-free DNA Origin**

**4. 1 Comparison of Two Library Preparation Methods for Low Input Methylomes**

Since the cell-free DNA in CSF is low abundance, we first tested two protocols for generating low-input methylomes on a single control sample (34109-1): Accel-NGS Methyl-Seq DNA Library Kit from Swift Biosciences and NxSeq Ampfree low DNA library kit from Lucigen. We found the Accel-NGS Methyl-Seq DNA Library Kit yielded superior and more even coverage of CSF cell-free DNA. We then used the Accel-NGS Methyl-Seq DNA Library Kit to prepare low input methylomes from all 4 control CSF samples and 1 patient CSF sample. Additionally, a library from unmethylated lambda phage genomic DNA was also prepared to serve as a control for the bisulfite non-conversion rate.

**4.2 Whole-Genome Bisulfite Sequencing (WGBS)**

Since we had a low amount of cell-free DNA input material, a significant quantity of adapter-dimers was generated following library preparation. Pronex beads were used to simultaneously deplete adapter dimers and enrich for library fragments. We sequenced 2 - 5 million paired-end reads for the four control CSF samples and one patient CSF sample. All samples, except one control, had close to or higher than 50% mapping rate, and low PCR duplicate numbers. All samples had 0.024x - 0.051x coverage for cytosines in the CG dinucleotide sequence context and 0.025x - 0.055x coverage for cytosines in the CH dinucleotide sequence context.

**4.3 Cell-of-Origin Analysis**

*4.3.1 Background* - Bray and colleagues described Kallisto (Bray *et al.*, 2016), an ultra-fast method that matches k-mers in raw sequencing reads to transcript specific k-mers using a hash table lookup. Cell-type specific DNA methylation patterns are analogous to gene sequence substrings (k-mers) that can delineate transcripts. Bisulfite sequencing produces sequencing reads with cell-specific k-mers that are introduced by the underlying DNA methylation patterns of cell subtypes contributing to the DNA pool analysed. Here, we convert cell-specific DNA methylation fractional measurements from WGBS data of reference cell-types to FASTA format from which DNA methylation k-mer’s are indexed and used to assign the cell-of-origin of single WGBS reads by hash table lookup.

*4.3.2 Public WGBS reference datasets* - whole genome DNA methylation profiles produced by WGBS of 11 cell-types provided reference datasets for our analysis. The cell-types included B-cell, CD14+ monocyte, CD34+ common myeloid progenitor, H1 and HUES64 embyronic stem cells, Natural Killer Cell, Spinal Cord, T-cell and Thyroid Gland produced by the ENCODE consortium (ENCSR284TCU, ENCSR017BUL, ENCSR388RMS, ENCFF601NBW, ENCSR354DMU, ENCSR334LSM, ENCSR334LSM, ENCSR458MAV, ENCSR663MXB, ENCSR601MHU). DNA methylation fractions stored in hg38.bed file format were cross-mapped to hg19 coordinates using CrossMap.py and the chain file hg38ToHg19.over.chain.gz and sorted using bedtools sort (Quinlan and Hall, 2010). In addition, we utilized our whole genome DNA methylation profiles (Lister *et al.*, 2013) from primary Central Nervous System (CNS) Neurons (NeuN+) and Glia (NeuN-) using WGBS aligned to hg19 reference genome.

*4.3.3 DNA methylation binarization and insertion into genomic context* – Fractional DNA methylation measurements for cytosines with good coverage (>=5 X) across all 11 cell-types were binarized using a threshold of 0.5 (DNA methylation fractions >=0.5 were assigned “C”, DNA methylation fractions <0.5 were assigned “T”) and split into forward (CT) and reverse (GA) stranded .bed files using split_bed.sh function. Each CT/GA.bed file was converted to .vcf format using bed_to_vcf.sh script and CT/GA.vcf files were sorted using Picard SortVcf (2019). The binarized DNA methylation of each cytosine (C/T) was inserted into their respective genomic position in FASTA format using GATK FastaAlternateReferenceMaker (Van der Auwera *et al.*, 2013) function and CT/ GA hg19 reference genomes made using bismark_genome_preparation (Krueger and Andrews, 2011). Note; we attempted to create a FASTA reference sequence from 32 tissues (ENCODE), however only 0.3% of cytosines were concordantly covered by all tissue’s, dramatically reducing the contiguous genomic regions that DNA methylation k-mers could be indexed. Therefore, a subset of 11 reference cell-types with good coverage and relevance to plasma and CSF derived cell-free DNA were selected.

*4.3.4 K-mer indexing* – Contiguous genomic regions with coverage across all 11 cell-types were selected from each cell-type CT/GA.fasta reference using bedtools getfasta (Quinlan and Hall, 2010). All cell-type CT/GA.fasta were concatenated into a combined FASTA reference of 123,744,456 contigues regions (5,624,748 genomic regions x 11 tissues x 2 reference sequences [CT/GA]) from chromosomes 1-7, X & Y, constituting 20.8% of total cytosines within the hg19 reference genome from which 1,035,106,323 sub-sequences (k-mers) were indexed using Kallisto index -k 31 (Bray *et al.*, 2016).

*4.3.5 Pseudoalignment of cell-free DNA WGBS reads from CSF and Plasma to cell-of-origin* – WGBS reads from all cell-free DNA samples (n=10) were trimmed using TrimGalore (--quality 30 --paired --phred33 --illumina --stringency 1 -e 0.1 --clip_R2 18 --three_prime_clip_r1 18 --gzip --length 20) and truncated using fastqutils truncate (read1=63bp, read2=49bp). WGBS reads were assigned (pseudoaligned) to cells-of-origin using Kallisto quant function (Bray *et al.*, 2016) by hash table lookup of the k-mer index described above. Sequencing reads from genomic regions lacking unique tissue-specific DNA methylation k-mers results in pseudoalignment to multiple cell-types and thus inflated counts (>type I errors) (**Supplementary** **Figure 4)**. To reduce Type I errors, uniquely pseudoaligned reads (assigned to only one cell-type) were selected and counted using indexing_and_quant.sh function. Note, due to the sex specificity of DNA methylation on the X and Y chromosomes, counts assigned to X/Y chromosones were removed from analysis. As expected, the number of uniquely pseudoaligned reads was correlated with sequencing depth (r=0.29, p= 0.002), therefore we normalised the counts by division with total unique pseudoaligned reads.


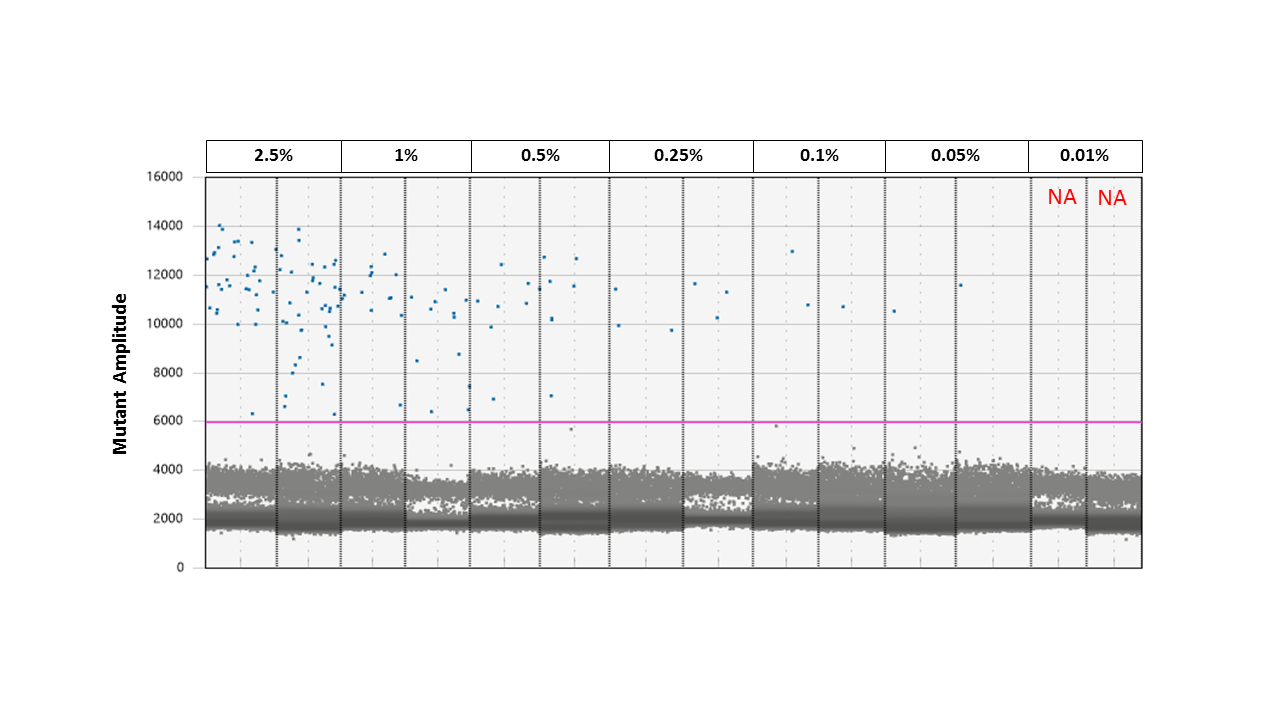


**Supplementary Figure 1. Limit of Detection for *LIS1* p.Lys64* ddPCR Assay**

Limit of detection of the ddPCR assay was established by serially diluting mutant samples into wild-type DNA in order to obtain different mutant/(mutant + wild-type) ratios: 2.5, 1, 0.5, 0.25, 0.1, 0.05 and 0.01%. An amplitude of 6000 was set as the positive mutant droplets threshold. Mutant allele at a frequency ≥ 0.25% could be consistently detected with at least three droplets.


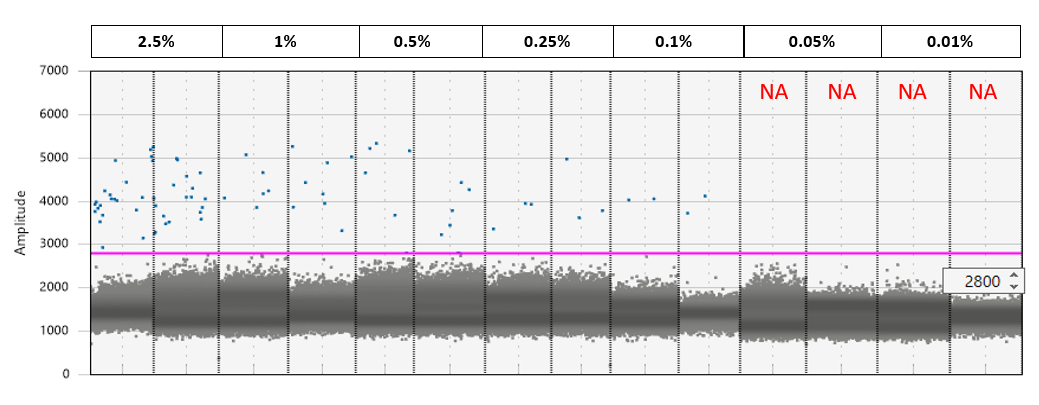


**Supplementary Figure 2. Limit of Detection for *TSC1* p.Phe581His*6 ddPCR Assay**

Limit of detection of the ddPCR assay was established by serially diluting mutant samples into wild-type DNA in order to obtain different mutant/(mutant + wild-type) ratios: 2.5, 1, 0.5, 0.25, 0.1, 0.05 and 0.01%. An amplitude of 2800 was set as the positive mutant droplets threshold. Mutant allele at a frequency ≥ 0.25% could be consistently detected with at least three droplets.


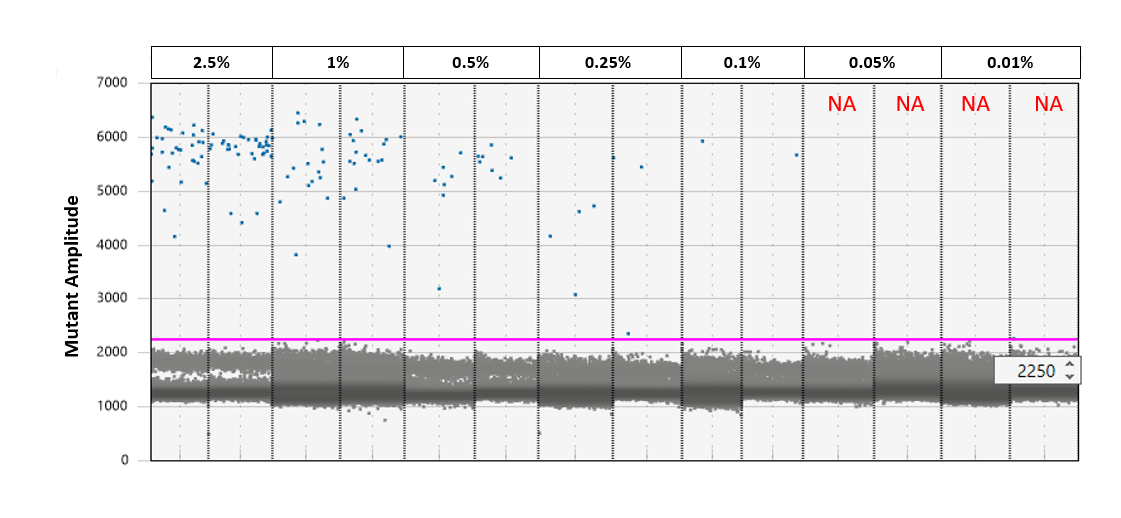


**Supplementary Figure 3. Limit of Detection for *BRAF* Val600Glu ddPCR Assay**

Limit of detection of the ddPCR assay was established by serially diluting mutant samples into wild-type DNA in order to obtain different mutant/(mutant + wild-type) ratios: 2.5, 1, 0.5, 0.25, 0.1, 0.05 and 0.01%. An amplitude of 2250 was set as the positive mutant droplets threshold. Mutant allele at a frequency ≥ 0.25% could be consistently detected with at least three droplets.

**
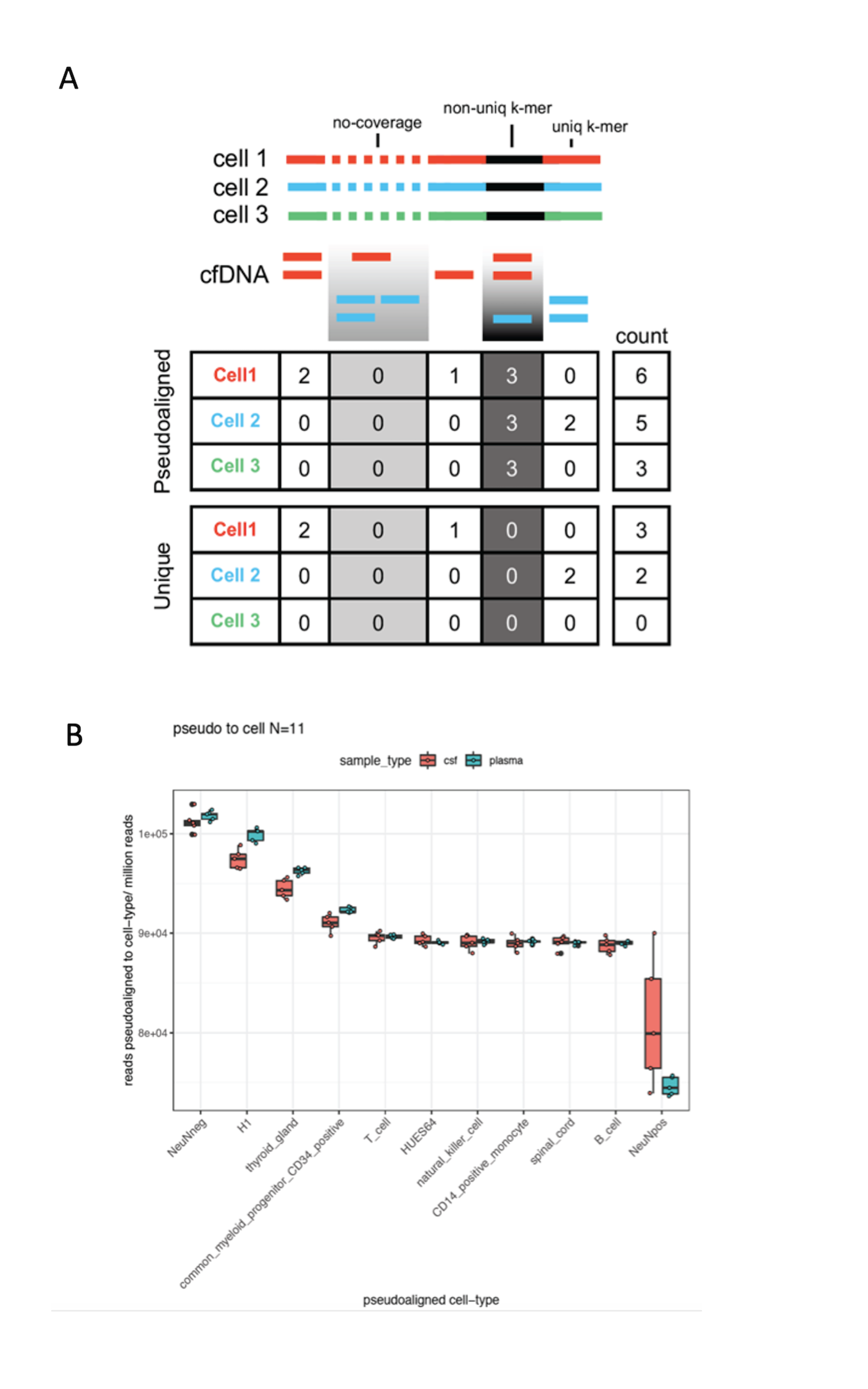
**

**Supplementary Figure 4. Pseudoalignment of WGBS cell-free DNA using DNA Methylation K-mers**

**A**. Schematic of DNA methylation k-mers indexing and pseudoalignment. DNA methylation k-mers are indexed from genomic regions with coverage across all reference cells/tissues, resulting in genomic regions within unique and non-unique cell/tissue-specific k-mers. Pseudoalignment of sequencing reads by hash table lookup of indexed k-mers assigns reads to each cell/tissue-type. The specificity of pseudoalignment can be improved by counting only sequencing reads uniquely pseudoaligned to a cell/tissue. **B**. Pseudoalignment of cell-free DNA from CSF and plasma to reference sequences/k-mers of 11 tissues.


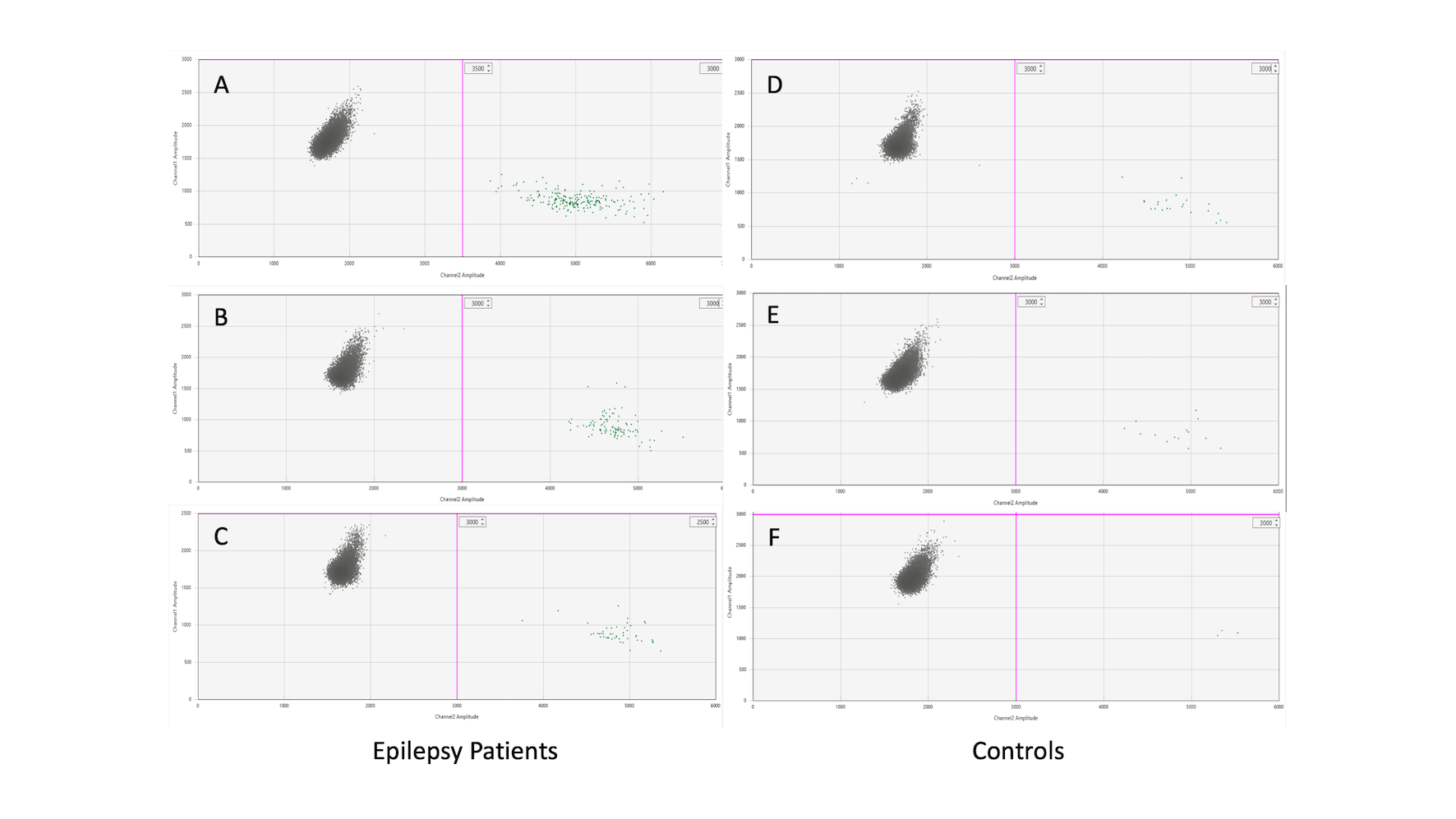
**Supplementary Figure 5. Examples of ddPCR output for CSF cell-free DNA quantitation**

Quantification of *GNAQ* wild-type copies in cell-free DNA from CSF of 3 epilepsy patients (A-C) and 3 controls without epilepsy (D-F) as a marker of CSF cell-free DNA levels. Green droplets contain a wildtype *GNAQ* wild-type copy and grey droplets are empty. The CSF cell-free DNA concentrations were computed as follows. Patients: A, 709.19 copies/mL; B, 492.2 copies/mL; C, 314.33 copies/mL in patients. Controls: D, 50.36 copies/mL; E, 40.09 copies/mL; F, 8.73 copies/mL.


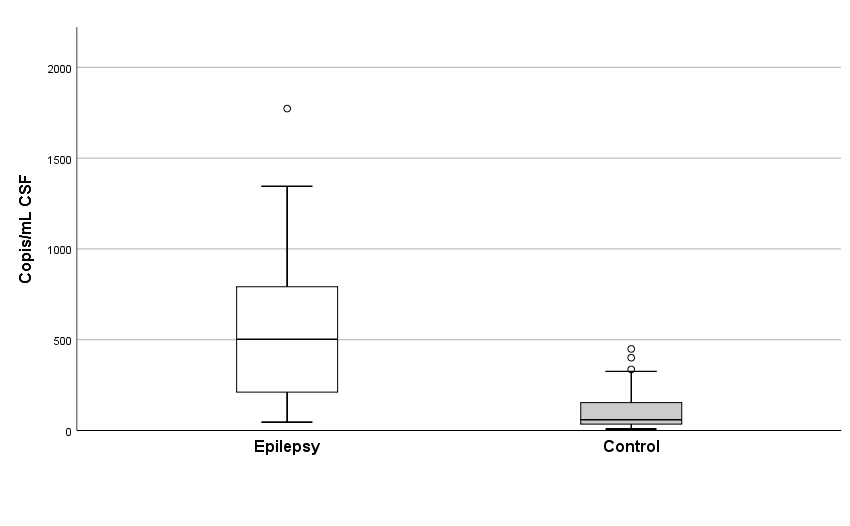


**Supplementary Figure 6. CSF cfDNA Concentration in Epilepsy Patients and Controls**

Horizontal lines show medians and interquartile ranges; open circles show outliers.

**
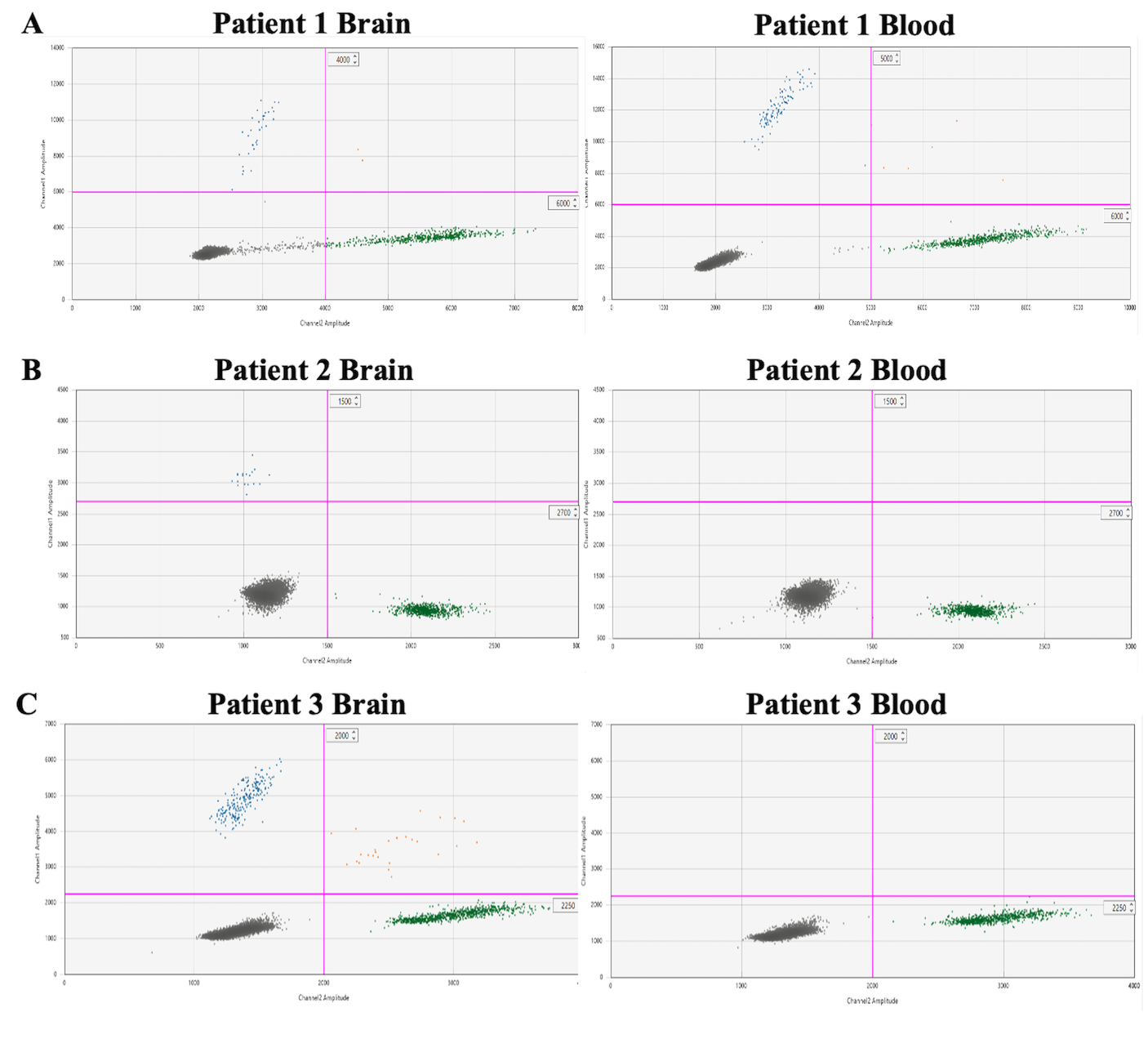
**

**Supplementary Figure 7. ddPCR 2D-plot for Brain and Blood in 3 Patients with Lesional Focal Epilepsy**

**A. 2D-plot showing droplets positive (blue, upper left quadrant) for *LIS1* Lys64* in patient 1.** Brain gDNA: mutant: 2.92 copies/ul; wild-type: 47.8 copies/ul; VAF: 5.8% (Damiano *et al.*, 2017). Blood gDNA: mutant: 10.1 copies/ul; wild-type: 59.7 copies/ul; VAF: 14.5%. **B**. **2D-plot showing droplets positive for *TSC1* p.Phe581His*6 in patient 2.** Brain gDNA: mutant: 2.03 copies/ul; wild-type: 85 copies/ul; VAF: 2.3%. Blood gDNA: mutant: 0 copies/ul; wild-type: 91.1 copies/ul. **C.** **2D-plot showing droplets positive for *BRAF* Val600Glu in patient 3**. Brain gDNA: mutant: 24.2 copies/ul; wild-type: 94.1 copies/ul; VAF: 20.4%. Blood gDNA: mutant: 0 copies/ul; wild-type: 75.4 copies/ul. Green droplets are wild-type copies, orange droplets are double-positive copies, and grey droplets are empty. VAF: variant allele frequency; gDNA: genomic DNA.

**Supplementary Table 1. Clinical and Genetic Information on the Patients with Epilepsy**

| **Patient ID** | **Age at CSF collection (years)** | **Sex** | **CSF Collection Method** | **Pathology** | **Brain somatic mutation** |
| --- | --- | --- | --- | --- | --- |
| 31225 | 2 | Male | Dural | Ganglioglioma | Suspect *BRAF* V600E |
| 31631 | 5 | Male | Dural | NA | No |
| 33049 | 2 | Female | Dural | FCD IIB | Known somatic *TSC1* c.1741_1742delTT |
| 33151 | 4 | Female | Dural | FCD I | No |
| 33296 | 4 | Female | Dural | FCD IIA | No |
| 33306 | 9 | Female | Dural | FCD IIB | No |
| 33357 | 4 | Male | Dural | FCD IIB | No |
| 33384 | 8 | Male | Dural | FCD IIB | No |
| 33427 | 12 | Male | Dural | HH | No |
| 33522 | 7 | Male | Dural | FCD IIB | No |
| 33523 | 4 | Female | Dural | FCD I | No |
| 33535 | 1 | Male | Dural | Ganglioglioma | No |
| 33601 | 16 | Female | Dural | FCD IIB | No |
| 33602 | 9 | Female | Dural | FCD IIA | No |
| 33761 | 6 | Male | Dural | FCD IIA | No |
| 33819 | 7 | Female | Dural | DNET | No |
| 33865 | 16 | Female | Dural | FCD IIB | No |
| 33947 | 7 | Female | Dural | FCD IIA | No |
| 33959 | 8 | Male | Dural | NA | No |
| 34045 | 7 | Female | Dural | FCD IIB | No |
| 34105 | 11 | Male | Dural | DNET | No |
| 34106 | 14 | Male | Dural | FCD IIA | No |
| 34107 | 1 | Female | Dural | FCD IIB | No |
| 34172 | 3 | Female | Dural | HH | No |
| T18648 | 6 | Male | Dural | NA | No |
| T26290 | 10 | Male | Dural | NA | No |
| 31783 | 18 | Female | Dural | FCD IIB | No |
| 34224 | 18 | Male | Dural | FCD IIB | No |
| 7354* | 41 | Female | Lumbar | Subcortical band heterotopia | Known mosaic *LIS1* K64X |

DNET: dysembryoplastic neuroepithelial tumour; FCD: focal cortical dysplasia; HH: hypothalamic hamartomas; NA: not available

*Patient 7354 (Patient 1 for the molecular diagnosis) is an additional patient to the 28 patients whose dural CSF was quantitated

**Supplementary Table 2: Primer and Probe Sequences and Annealing Temperatures for ddPCR Assays**

| Assay | Sequence | | Tm (°C) |
| --- | --- | --- | --- |
| *GNAQ*  Arg183Gln | Commercial assay (Bio-Rad 10049047), sequence not available | | 55 |
| *LIS1*  Lys64* | Primer F | TGGAAAAAAAATGGA | 55 |
|  | Primer R | TGCAGAAGAATGTTATTTTCAGAA |  |
|  | WT probe | VIC-ATTACAAAAGAAGGTAACTAA-MGB-NFQ |  |
|  | MUT probe | FAM-ATTACAAAAGTAGGTAACTAA-MGB-NFQ |  |
| *TSC1* Phe581His*6 | Primer F | GTCGGAGGTGGAATTTTACAAGGA | 58 |
|  | Primer R | GGGAATGCCAGACTTCTTTGGA |  |
|  | WT probe | VIC-CCAGTATCTTCACTCCC-MGB-NFQ |  |
|  | MUT probe | FAM-CCAGTATCCACTCCC-MGB-NFQ |  |
| *BRAF*  Val600Glu | Commercial assay (Bio-Rad dHsaCP2000027 and dHsaCP2000028), sequence not available | | 55 |

WT: wild-type; MUT: mutant

**Supplementary Table 3. CSF cell-free DNA Concentration in Epilepsy Patients and Controls**

| Group | Number | Methods of CSF collection | Age of CSF collection  (years, mean [median]) | CSF cell-free DNA concentration  (copies/mL CSF, median) | Estimated CSF cell-free DNA concentration  (ng/mL CSF, median)* |
| --- | --- | --- | --- | --- | --- |
| Epilepsy | 28 | Dural puncture | 7.8 [7] | 502 | 1.5 |
| Control | 28 | Lumbar puncture | 46.9 [47] | 61 | 0.18 |

* CSF cell-free DNA in ng/mL CSF is estimated based on the mass of 1 copy of haploid human genome is 0.003 ng

**Supplementary Table 4. ddPCR Raw Data of the 3 Patients with Focal Epilepsy and Brain Malformations**

| **Patient** | **Sample**  **Type** | **Mutant**  **Copies/20** μl **well** | **Wild-type**  **Copies/20** μl **well** | **Droplets positive for Mutant** | **Droplets positive for Wild-type** | **Accepted Droplets** | **Fractional**  **Abundance (%)** |
| --- | --- | --- | --- | --- | --- | --- | --- |
| **Patient 1**  **(*LIS1*)** | CSF (Test 1) | 35.7 | 324 | 19 | 171 | 12521 | 9.95 |
|  | CSF (Test 2) | 36.4 | 375 | 17 | 174 | 11002 | 8.84 |
|  | Brain* | 58.4 | 956 | 35 | 562 | 14113 | 5.76 |
|  | Blood | 202 | 1193 | 118 | 682 | 13792 | 14.5 |
| **Patient 2**  **(*TSC1*)** | CSF | 9.48 | 112 | 4 | 47 | 9929 | 7.83 |
|  | Brain | 40.6 | 1700 | 18 | 728 | 10443 | 2.33 |
|  | Blood | 0 | 1823 | 0 | 670 | 8987 | / |
| **Patient 3**  **(*BRAF*)** | CSF | 8.38 | 254 | 5 | 151 | 14047 | 3.19 |
|  | Brain | 484 | 1882 | 223 | 880 | 11446 | 20.4 |
|  | Blood | 0 | 1508 | 0 | 739 | 11902 | / |

* Damiano, J.A., et al. 2017

**ADDITIONAL REFERENCES**

Pan W, Gu W, Nagpal S, Gephart MH, Quake SR. Brain tumor mutations detected in cerebral spinal fluid. Clinical chemistry 2015; 61(3): 514-22.

Uchiyama Y, Nakashima M, Watanabe S, Miyajima M, Taguri M, Miyatake S, et al. Ultra–sensitive droplet digital PCR for detecting a low–prevalence somatic GNAQ mutation in Sturge–Weber syndrome. Sci Rep. 2016;6:22985.

Rowlands V, Rutkowski A , Meuser E , Carr TE, Harrington EA , Barrett JC. Optimisation of robust singleplex and multiplex droplet digital PCR assays for high confidence mutation detection in circulating tumour DNA. Sci Rep 2019; 9(1):12620.

Picard Toolkit.: GitHub Repository. <http://broadinstitute.github.io/picard/> ; Broad Institute; 2019.

Bray NL, Pimentel H, Melsted P, Pachter L. Near-optimal probabilistic RNA-seq quantification. Nature biotechnology 2016; 34(5): 525-7.

Krueger F, Andrews SR. Bismark: a flexible aligner and methylation caller for Bisulfite-Seq applications. Bioinformatics 2011; 27(11): 1571-2.

Lister R, Mukamel EA, Nery JR, Urich M, Puddifoot CA, Johnson ND*, et al.* Global epigenomic reconfiguration during mammalian brain development. Science (New York, NY) 2013; 341(6146): 1237905.

Quinlan AR, Hall IM. BEDTools: a flexible suite of utilities for comparing genomic features. Bioinformatics (Oxford, England) 2010; 26(6): 841-2.

Van der Auwera GA, Carneiro MO, Hartl C, Poplin R, Del Angel G, Levy-Moonshine A*, et al.* From FastQ data to high confidence variant calls: the Genome Analysis Toolkit best practices pipeline. Curr Protoc Bioinformatics 2013; 43: 11 0 1- 0 33.
